# Crossing Death Valley: Bringing Neurotechnology to Psychiatric Clinics in Alberta, Canada

**DOI:** 10.3389/fpsyt.2020.00135

**Published:** 2020-03-06

**Authors:** Frank P. MacMaster, Nick Mitchell, Allison Bichel, Marni Bercov, Gayle Thompson, Victoria Suen, Allison Strilchuk, Katherine Rittenbach

**Affiliations:** ^1^Department of Pediatrics and Psychiatry, Cumming School of Medicine, University of Calgary, Calgary, AB, Canada; ^2^Strategic Clinical Network for Addictions and Mental Health, Alberta Health Services, Calgary, AB, Canada; ^3^Department of Psychiatry, University of Alberta, Edmonton, AB, Canada; ^4^Strategic Clinical Network for Addictions and Mental Health and Maternal, Newborn, Child & Youth Strategic Clinical Network, Alberta Health Services, Calgary, AB, Canada; ^5^Department of Nephrology, Cumming School of Medicine, University of Calgary, Calgary, AB, Canada; ^6^Strategic Clinical Network, Alberta Health Services, Calgary, AB, Canada

**Keywords:** depression, health policy, knowledge translation, transcranial magnetic stimulation, treatment resistant depression

## Abstract

Depression is a major public health problem, with a lifetime and 12-month prevalence estimated at 18 and 6% of adults. Depression is costly in terms of treatment and lost productivity and is the main burden of mental illness across the globe. Existing pharmacological and psychological treatments for depression result in clinically meaningful improvements in <60% of patients. An emerging treatment approach is non-invasive brain stimulation of depression-related brain targets through transcranial magnetic stimulation (TMS). In this perspective, we detail our efforts on bringing TMS to clinical populations in Alberta by utilizing a novel organizational structure that bridges the gap between academia and the health care system. The Addictions and Mental Health Strategic Clinical Network worked with stakeholders to (1) examine the evidence, (2) develop clinical tools for patient selection and protocol application, (3) create overall implementation and evaluation plans to aid in further scale and spread, and even (4) fund the purchase and deployment of devices. Through this work, five publicly supported clinics now exist in Alberta.

## Introduction

Psychiatry in Canada has not benefitted broadly from advances in technology since the advent of electroconvulsive therapy (ECT) over three quarters of a century ago (1). The gap between bench and bedside in Canada has been referred to as a “Death Valley” and has plagued the application of innovative research to improve the lives of Canadians (2). The process of translating discoveries into treatments is slow, costly, and often unsuccessful—with most being shelved before their benefit is realized (3). Adoption of innovation can also face particular challenges under a single payer system (4). Here, we detail our perspective on bringing transcranial magnetic stimulation (TMS) to clinical populations in Alberta by utilizing a novel organizational structure that bridges the gap between academia and the health care system.

## What is Depression and how do we Treat it now?

Clinical depression (or major depressive disorder) is characterized by a persistent sadness, a loss of interest in activities that the person normally enjoys doing, and an impairment in daily functioning that last at least 2 weeks (5). More than 300 million people worldwide suffer from clinical depression (referred to as depression going forward), it is the leading cause of disability worldwide, and is a major contributor to the global burden of disease (6). The causes for depression are not well-understood, but some hypothesized pathophysiological mechanisms of depression include altered neurotransmission, hypothalamic-pituitary-adrenal (HPA) axis abnormalities involved in chronic stress, inflammation, reduced neuroplasticity, and network dysfunction (7).

Current care practices for depression target response (acute treatment) and maintenance (8). This is achieved typically through the use of antidepressant medication, psychotherapy like cognitive behavioral therapy (or CBT), and/or ECT. According to Canadian Network for Mood and Anxiety Treatments (CANMAT) guidelines, selective serotonin reuptake inhibitors, and serotonin and noradrenaline reuptake inhibitors should be used as first-line antidepressant treatments (9, 10).

However, depression is a heterogeneous disorder and no one treatment works for all patients. Frontline treatments for depression are not effective in 20–60% of patients, and success rates vary depending on the treatment used (11, 12). This leaves a large gap in care, as clients with depression that does not respond to first line treatment may have treatment resistant depression (TRD). As there is no consensus-based definition for treatment-resistant depression, we undertook a systematic review and interviews with key Canadian informants to establish one (13)—with two treatment failures being the most common definition being endorsed. Treatment must be considered adequate, but considerable variation exists for how to define adequate (13). ECT can be an effective treatment for treatment-resistant depression but is often considered only as a last resort due to fear of side effects and stigma (14). Hence, there is space for an intervention such as TMS before ECT is considered. Using our definition (13) and Alberta Health Services (AHS) administrative data, we conservatively estimate that there are over 54,000 individuals with treatment-resistant depression in Alberta aged 12 years and up (15). The vast majority of these people with treatment-resistant depression do not receive ECT however, and become trapped in a gap, failing to receive effective care.

This failure to improve depressive symptoms comes at a cost to the system as well. We analyzed data from the entire population of Alberta, Canada from January 1, 2015 to December 31, 2017 inclusive (15). We identified a sub-cohort of people who were receiving 3 or more antidepressants or augmentation medications within 90 days or individuals who had two different medications within 90 days and a series of inpatient or outpatient electroconvulsive therapy (ECT) for depression treatment as being TRD and examined health care utilization over the selected time period. Our data shows the median cost to the health care system of treatment-resistant depression is 3 times that of depression and 9 times that of a typical Albertan, with a mean cost of $24,317 per case of treatment-resistant depression (15) (report available upon request from the corresponding author). By definition, people with treatment-resistant depression are still ill despite this investment. Hence, we are spending much more for treatment-resistant depression without providing relief to the patients.

## What is Transcranial Magnetic Stimulation (TMS)?

TMS uses electromagnetic induction to deliver an electric signal to the human brain (16). An electric current is briefly applied to a stimulator coil to produce a rapidly changing magnetic field. This induces a flow of electric current in nearby conductors, including cortical neurons. TMS has been used both as a probe of brain function and as a clinical intervention for patients experiencing major depressive disorder. TMS was first used as a treatment for depression over two decades ago by George et al. (17). It has since become an accepted tool in the treatment of depression and is considered a first-line intervention for adults failing at least one trial of an antidepressant according to CANMAT guidelines (18). Although TMS is a proven treatment technology and one that is commonly used in other jurisdictions (e.g., the United States), it has not been widely adopted in Canada. TMS does offer an opportunity to help close the treatment gap detailed above and better serve patients with treatment-resistant depression. The need is there, and so is the technology to address it. TMS may also be cost-effective, as TMS results in both a reduction in health care service utilization (19) and increase in quality-adjusted life years (QALYs) (8).

The challenge for care providers has been to identify ways to expand patient access to TMS. The main barriers generally being a lack of a local champion (person or organization) and the lack of a clear path for bringing evidence to the clinic.

## History of TMS in Alberta

**Figure 1:**
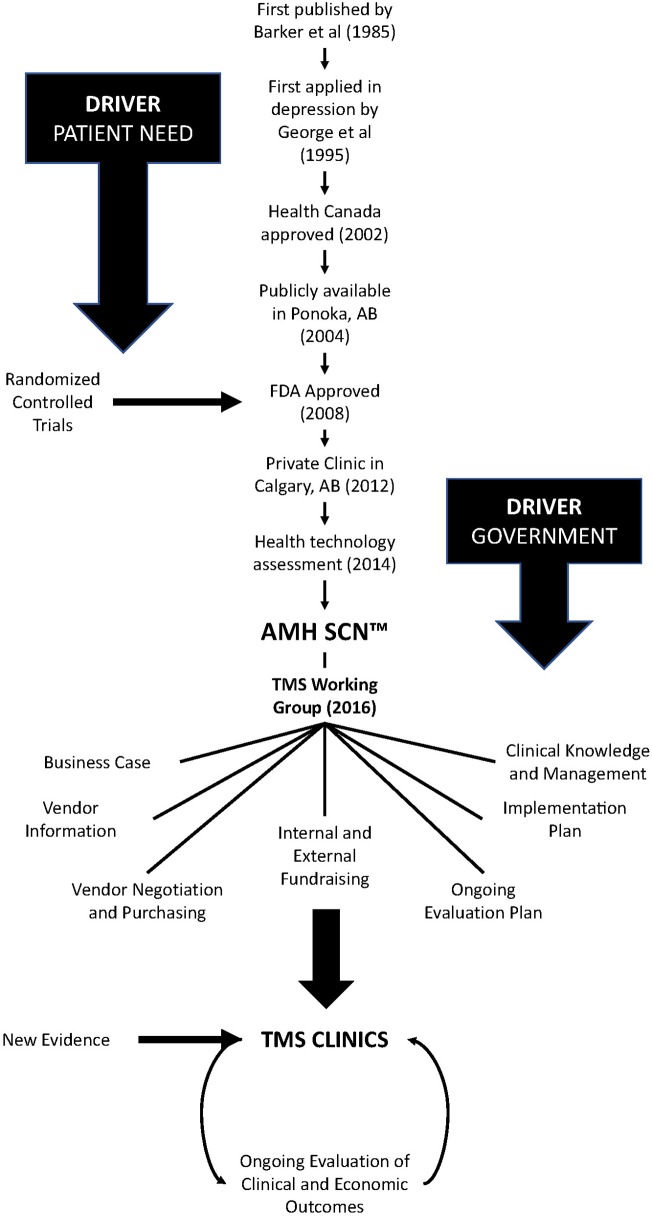
Schematic showing progress of transcranial magnetic stimulation (TMS).

TMS was approved by Health Canada 17 years ago, and by the United States Food and Drug Administration afterward (see Figure 1). In Alberta, a publicly funded service was first opened in Ponoka in 2004 and a private clinic was added in Calgary in 2012. In 2013, research began in earnest in Alberta at the Alberta Children's Hospital and the University of Calgary to evaluate TMS as a treatment for youth with depression (20). The research involved in that open-label trial (NCT01731678) (20) provided first-hand experience and data on patient outcomes and the safety, tolerability, and acceptability of TMS as a treatment for depression in youth. This work, combined with the clinical work in Ponoka, were the critical first steps in developing a plan to create a TMS clinical service for treatment of depression in Alberta.

## Moving From Bench to Bedside

Around this time (2013), the Government of Alberta, initiated a Health Technology Assessment (HTA) (8) to review and assess whether transcranial magnetic stimulation (TMS) should become a publicly funded service in Alberta for people with treatment-resistant depression. The HTA Unit, along with an Expert Advisory Group comprising clinicians and scientists with experience in TMS, released a report in 2014 that provided evidence supporting the use of TMS for depression as well as initial estimates of treatment costs (8).

## Actively Engaging Stakeholders

The next step involved getting the appropriate partners and stakeholders together to develop an implementation strategy. Alberta Health engaged the Addiction and Mental Health Strategic Clinical Network™ (SCN™) to lead this initiative and work closely with all stakeholders to design and test an implementation strategy for TMS. SCNs have been part of Alberta's health system since 2012 and focus on driving health care improvements through research and innovation (21, 22). SCNs serve a critical role in translational research by identifying gaps in care, opportunities to standardize care pathways, and chances to bring evidence into practice by leveraging relationships between the government, clinicians, academic researchers, policymakers, people with lived experience, and the public. The goal of SCNs are to identify and test innovative ways of delivering care to optimize patient outcomes, experience and value and to support more efficient and integrated knowledge translation across the health system.

The Addiction and Mental Health SCN created a working group comprising clinicians, operational managers, people with lived experience, and researchers from across the province, to lead the TMS evaluation and serve as a decision-making hub. The working group helps Alberta Health Services bridge the research-to-practice divide. Researchers, clinicians, and operational leads have different frames of reference about what matters and getting everyone in the same room to debate options, resolve potential conflicts, and clarify priorities resulted in a galvanized path forward.

The SCN also worked closely with operational stakeholders, including Contracting, Procurement and Supply Management (CPSM) to develop a Request for Information from potential TMS vendors. This tool was used to inform the business case for TMS and develop a purchasing process for TMS devices (23). The Addiction and Mental Health SCN convened a subgroup of knowledgeable clinicians and researchers to guide this process. Vendors submitted responses to the purchasing call and the SCN working group selected a vendor in 2018. By having a single vendor, we were able to lower costs and implement common training practices across sites. Naturally, the risk of choosing a single vendor is that if that company goes out of business, ongoing maintenance and support become problematic. Variations in machine capabilities could also be considered a concern under a single vendor, but the goal is to achieve high protocol fidelity and actually limit variation.

Another practical consideration is impact on space. The footprint of TMS is not large (space for the chair and machine) and can fit into most clinical rooms with relative ease. Some sound proofing or adjustment of the room's electrical power capabilities are sometimes required (and come with a financial cost). Dedicated TMS rooms do come with an opportunity cost for the hospital as they preclude use of that space in other ways.

## Developing a Viable Business Case

In developing a business case for TMS in Alberta, the Addiction and Mental Health SCN explored a number of approaches to implementation and evaluated their viability and potential cost to the health system (23) (available upon request from the corresponding author). Using criteria that aligned with health care quality objectives, the group endorsed the approach that addressed a triple aim of better health, better care, and lower costs. One potentially controversial decision was to not add a physician billing code specifically for TMS. Instead, psychiatrists involved in the assessment and care of patients receiving TMS are remunerated using the existing billing codes (i.e., those for providing consultation and ongoing treatment). This model sees psychiatrists as experts overseeing a clinical service that is provided by a multidisciplinary team. Further, it serves as an example of the type of “innovation” and creative thinking that may be required to advance similar health care implementation objectives (4). In this case, the SCN felt an additional physician-related expense would skew the cost curve for TMS to an extent that would render broad implementation unlikely. A potential concern with not adding a physician billing code is that this may limit physician uptake or sustainability.

## Measurement, Reporting, and Further Navigating Death Valley

Determining a standard clinical approach for TMS, and how best to measure and evaluate its success, were key challenges the Addiction and Mental Health SCN faced in developing its implementation strategy. The group used the AHS Clinical Knowledge Clinical Management (CKCM) process to benchmark and synthesize best practices and treatment guidelines (24), such as those established by the Canadian Network for Mood and Anxiety Treatments (CANMAT) (18). Initially, there were different perspectives and a level of conflict about a standard clinical approach. The CANMAT guidelines were very timely and helped resolve the debate.

The CKCM Service includes a flexible, evidence-informed monitoring program that will enable the Addiction and Mental Health SCN to evaluate and incorporate changes into the plan as evidence developed for other treatment protocols (i.e., theta burst, novel target sites). Understanding the importance of continued research and evaluation as an essential part of any implementation strategy—whether it be an innovative treatment technology or new model of care—the Addiction and Mental Health SCN adopted an evidence-based approach from the beginning and has strived to make data collection and analysis an integrated part of care delivery.

Partnering with the CKCM Service was critical to the success of this initiative. The CKCM team documented an ongoing evaluation plan in which treatment outcomes (i.e., clinical response as measured by the Hamilton Depression Rating Scale) and protocol fidelity could be monitored, ensuring consistency in practice and clear data. This plan also highlighted who should run the TMS machines and determined that technicians could run the protocols with medical oversight by a nurse. Going forward, ongoing provincial collaboration will be important to leverage and support human resource and training requirements, ensure protocol fidelity and consistency in care, and continue to evaluate outcomes and develop evidence-based care pathways.

At this time of this writing, Alberta has opened two new TMS clinics in Edmonton and two in Calgary, with more slated to follow. This milestone is a direct result of the Addiction and Mental Health SCN and its partners' commitment and persistence and comes 15 years after the first public clinic opened in Ponoka, Alberta in 2004.

## Discussion

To truly impact depression in the province, the scale and spread of treatments and interventions must be adequate to the challenge. Assuming that a TMS machine services ~200 people in a year (the time it takes to deliver the standard protocol as described in the CKCM document), it would take more than 100 TMS machines to meet the current demand. The standard 37.5 min protocol over 5 days a week for 6 weeks is almost 19 h of treatment for an individual (18). Considerable investment would be required to provide treatment on this scale, ~$9 million in capital funds to cover the cost of the machines alone. Although this figure seems large, it represents about 0.1% of the cost of depression to the Alberta economy.

The task ahead is to scale implementation of TMS across the province, quantify and report outcomes (i.e., clinical response, protocol fidelity), and use this data to (i) demonstrate both the benefits realized by patients and economic benefits (i.e., health care utilization costs of TMS treated patients compared with TRD patients not receiving TMS); (ii) evaluate and refine care pathways, and (iii) assess the opportunity for further spread. As above, the involvement of key stakeholders (i.e., government, clinicians, academic researchers, policymakers, people with lived experience, and the public) will be critical.

It is critical to note that this approach worked for our specific jurisdiction within the Canadian health care system. Specific aspects of our model of implementation selected were designed to meet those particular conditions and may have different ramifications if applied under other jurisdictions. For example, our decision to not use a direct physician billing model might not work in other jurisdictions. The overall framework of engagement and building a case for support are broadly applicable though.

Even with enthusiasm generated by the Naylor Report (25) and basic science generated innovations, medicine—not just psychiatry—has been poor at realizing those innovations into the clinic. While there should be barriers to moving unproven, novel discoveries to the clinic (3), this should not be the case for a proven treatment technology like TMS that is widely used in other jurisdictions.

## Author Contributions

All authors contributed to the drafting, editing, and revising of the manuscript and its contents.

### Conflict of Interest

The authors declare that the research was conducted in the absence of any commercial or financial relationships that could be construed as a potential conflict of interest.
